# Hospitalizations of Adolescents with Psychiatric Disorders in a Pediatric Unit: A 10-Year Retrospective Study

**DOI:** 10.3390/pediatric18040111

**Published:** 2026-08-10

**Authors:** Francesco Accomando, Melodie O. Aricò, Enrico Valletta

**Affiliations:** Pediatrics Department, G.B. Morgagni–L. Pierantoni Hospital, Azienda Unità Sanitaria Locale (AUSL) Romagna, 47121 Forli, Italy; melodieolivialoredanarosa.arico@auslromagna.it (M.O.A.); enrico.valletta@auslromagna.it (E.V.)

**Keywords:** adolescents, mental health, hospitalization, suicidal ideation, eating disorders

## Abstract

**Background:** To assess temporal trends in psychiatric hospitalizations among adolescents admitted to a general pediatric ward in Forlì, Italy, between 2016 and 2025 and to examine diagnostic patterns and their association with sex and age, using the individual patient as the primary unit of analysis. **Methods:** Single-center retrospective study including all hospitalizations for psychiatric disorders in patients aged 10–17 years over a 10-year period. Each discharge episode was assigned to a single dominant diagnostic category. The primary analyses were conducted at the level of the individual patient (first admission), while episode-level analyses were retained as a pre-specified sensitivity analysis reflecting inpatient burden. **Results:** A total of 165 adolescents (210 hospitalization episodes) were included; mean (SD) age was 14.47 (1.73) years, and females were 77% of patients (80.5% of episodes). Admissions increased significantly over time (patients: IRR 1.20 per year, 95% CI 1.13–1.27; *p* < 0.001), with 124/165 (75.2%) patients first admitted in 2021–2025. Suicidal ideation/attempt (27.3%) and eating disorders (15.2%) were the most frequent diagnoses, and both showed a significant upward trend. Overall, diagnosis was associated with sex (*p* = 0.004): most diagnoses, including the two most frequent, were female-predominant, whereas psychomotor agitation was over-represented in males. Age at admission increased modestly over time and was higher in 2021–2025. Twenty-seven patients accounted for 45 readmissions, concentrated in the most severe diagnoses. **Conclusions:** Psychiatric hospitalizations of adolescents in a general pediatric ward rose substantially over the decade, especially from 2021, driven mainly by suicidal ideation/attempt and eating disorders. Findings were robust to analysis at the patient level and support the role of the general pediatric ward as a sentinel setting for severe adolescent mental distress.

## 1. Introduction

In recent years, adolescent mental health has become an increasingly important public health priority. According to the World Health Organization, about one in seven adolescents aged 10–19 years lives with a mental disorder, and UNICEF has highlighted the broad impact of mental health problems on the well-being of children and adolescents [1,2]. In this context, hospitalization may be regarded as a marker of the most severe forms of psychological distress, because it captures patients with the greatest clinical, organizational, and psychosocial complexity.

International studies have documented a progressive increase in mental-health-related hospitalizations and acute-care presentations, including emergency department visits, among young people, with further worsening during and after the COVID-19 pandemic [3,4,5,6]. Increases have been reported for depression, anxiety, suicidality, self-harm, and eating disorders, with a disproportionate impact on female adolescents and older age groups [4,5,7,8]. In Italy, several reports have described increasing psychological vulnerability in adolescence and mounting pressure on child and adolescent neuropsychiatry services [9,10,11,12,13].

However, most available data derive from dedicated child and adolescent psychiatric units, whereas longitudinal data from general pediatric wards remain limited. This distinction is not merely descriptive: where inpatient neuropsychiatric beds and community resources are scarce, general pediatric wards increasingly absorb the acute psychiatric demand that cannot be accommodated elsewhere. Trends observed in such a setting may therefore provide a useful sentinel measure of the most severe component of adolescent mental distress, while also quantifying a burden that is often invisible in psychiatry-based statistics. Against this background, the aim of the present study was to describe the evolution of psychiatric hospitalizations among adolescents admitted to a general pediatric ward over a 10-year period (2016–2025), focusing on diagnostic, sex, and age distribution and on pre- versus post-2020 changes, while adopting the individual patient as the primary unit of analysis.

## 2. Methods

### 2.1. Study Design and Setting

We conducted a retrospective observational study of psychiatric hospitalizations occurring in the Pediatrics Unit of G.B. Morgagni–L. Pierantoni Hospital, Forlì, Italy, between January 2016 and December 2025. The ward admits patients from the neonatal period through adolescence (0–17 years) and represents the main hospital referral center for acute pediatric conditions in a district area of about 200,000 inhabitants. The study was designed and reported in accordance with the STROBE guidelines for observational studies.

### 2.2. Participants and Data Sources

We included all hospitalizations for psychiatric disorders among patients aged 10–17 years at admission. Although adolescence is conventionally defined by the World Health Organization as 10–19 years [1], patients aged 18 years or older were not included because the local pediatric ward admits patients only up to 17 years of age. Age at admission was calculated as the interval between date of birth and date of admission. Data were extracted from the hospital discharge database, which systematically records information on each admission episode; for each episode we retrieved an anonymized patient identifier, sex, date of admission, age at admission, and the recorded discharge diagnosis. All data were anonymized before analysis. No missing data were present for the variables used in the analyses (sex, age, year of admission, and diagnostic category were complete for all 210 episodes). Annual counts of all admissions to the pediatric ward were obtained from hospital administrative records and used as denominators for the rate-based analyses.

### 2.3. Diagnostic Classification

Each episode was assigned to a single main clinical category based on the prevailing condition responsible for admission and inpatient care. When multiple diagnoses were present, classification was based on the clinical condition judged to be dominant in determining the need for hospitalization. The categories were suicidal ideation/attempt, eating disorders, psychomotor agitation, anxiety/panic disorder, mood disorder, conduct/behavior disorder, non-suicidal self-harm, alcohol/substance use, conversion/functional disorder, and psychosis/delirium. Episodes classified as suicidal ideation/attempt frequently showed psychiatric comorbidities such as mood or anxiety disorders; to preserve mutual exclusivity, each episode was assigned only to the category considered primarily responsible for admission. Initial classification was performed by one investigator based on the recorded discharge diagnosis. Cases considered ambiguous were discussed among all authors, and the final classification of all records was reviewed and approved by all authors.

### 2.4. Unit of Analysis and Outcomes

Because a minority of adolescents were hospitalized more than once during the study period, treating every episode as independent would violate the assumption of independence and could inflate statistical significance. Therefore, the primary analyses used the individual patient as the unit of analysis, retaining the first admission of each patient. Episode-level analyses, in which repeated admissions were counted individually, were retained as a pre-specified sensitivity analysis and interpreted as a measure of inpatient burden rather than of patient-level epidemiology. The primary outcome was the annual number and trend of hospitalizations. The corresponding annual rate per 1000 total pediatric admissions was examined in an additional analysis accounting for annual ward activity. Secondary outcomes were the diagnostic distribution, overall and diagnosis-specific temporal trends, the association between diagnosis and sex, age differences across diagnostic groups, the temporal trend in age at admission, and the comparison between the two 5-year periods 2016–2020 and 2021–2025.

### 2.5. Statistical Analysis

Analyses were performed in Python (version 3.11) using the statsmodels, scipy, and pandas libraries. Temporal trends in annual counts, overall and by diagnosis, were modeled using Poisson regression, with results reported as incidence rate ratios (IRRs) per year with 95% confidence intervals (95% CIs); diagnosis-specific trends were adjusted for multiple testing using the false discovery rate (FDR, Benjamini–Hochberg). To account for annual variation in overall pediatric ward activity, the overall temporal trend was additionally assessed using Poisson regression with the natural logarithm of annual total pediatric admissions as an offset. Because overdispersion was present, standard errors and confidence intervals were scaled using the Pearson chi-square statistic. The association between diagnosis and sex was assessed with the chi-square test and quantified with Cramér’s V; because several diagnostic categories were small, cells with low expected counts were present and the corresponding estimates are interpreted with caution. To explore whether sex patterns differed between the two 5-year periods, diagnostic categories were additionally cross-tabulated by sex and period at the patient level; owing to sparse cells, this analysis was considered descriptive. Age differences across diagnoses were assessed with the Kruskal–Wallis test. The relationship between age and year of admission was evaluated with ordinary least squares (OLS) regression and Spearman correlation. For four pre-specified diagnoses (suicidal ideation/attempt, eating disorders, psychomotor agitation, and mood disorder), multivariable logistic regression models were fitted with sex, age, and year (centered on 2016) as predictors; results are reported as odds ratios (ORs) with 95% CIs and McFadden’s pseudo-R^2^, with the number of events per model reported to allow assessment of model stability. The two 5-year periods were compared for sex (chi-square) and age (Mann–Whitney), and annual and period-specific admission rates per 1000 total pediatric admissions were computed. All tests were two-sided, and *p* < 0.05 was considered statistically significant. Computations were cross-checked for internal consistency; artificial-intelligence tools were used only for language editing and did not perform the statistical analysis.

## 3. Results

### 3.1. Cohort and Overall Trend

**Figure 1:**
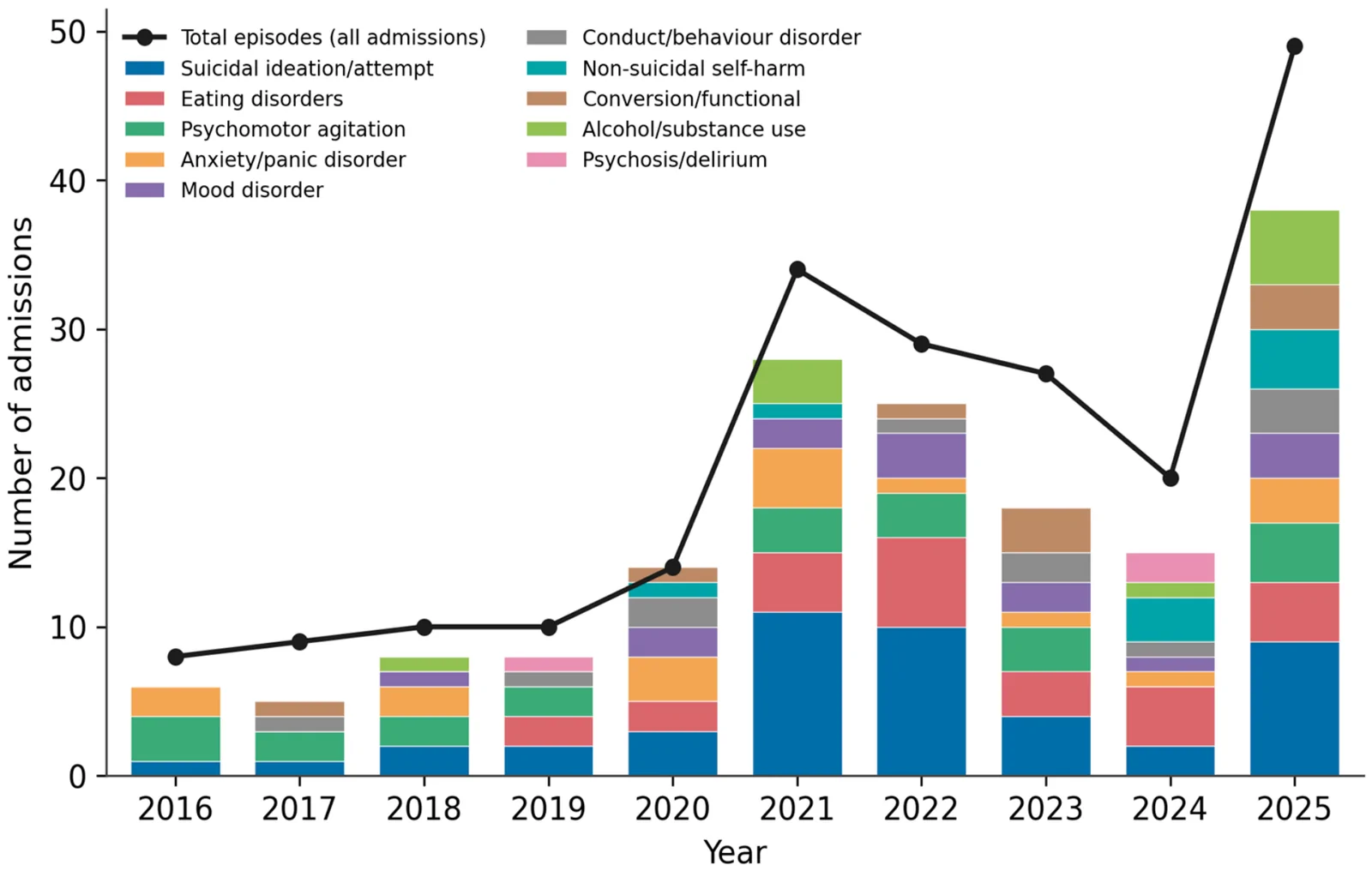
Annual number of admissions stratified by diagnosis (unique patients, stacked bars) with the total number of hospitalization episodes per year (black line).

From a database of 9090 pediatric hospital admissions recorded between 2016 and 2025, 210 psychiatric hospitalizations of adolescents were identified, corresponding to 165 individual patients. Twenty-seven patients were hospitalized more than once during the decade, accounting for 45 repeated admissions. In the primary (patient-level) analysis, females represented 77% of the cohort (80.5% of episodes) and the mean (SD) age was 14.47 (1.73) years (median 15, IQR 13–16; range 10–17). The number of admissions increased progressively over the study period, with a marked rise from 2021 onward (Figure 1). Poisson regression confirmed a significant temporal increase, with an IRR per year of 1.20 (95% CI 1.13–1.27; *p* < 0.001) at the patient level and 1.20 (95% CI 1.14–1.27; *p* < 0.001) at the episode level. Annual total pediatric admission volumes ranged from 672 to 1093. After accounting for this variation, the temporal increase remained significant, with an IRR of 1.17 per year at the patient level (95% CI 1.04–1.32; *p* = 0.011) and 1.17 at the episode level (95% CI 1.05–1.30; *p* = 0.003). Annual denominators, psychiatric admission counts, and corresponding rates are reported in Supplementary Table S1, and 124/165 (75.2%) patients were first admitted in the 2021–2025 period.

### 3.2. Diagnostic Distribution and Diagnosis-Specific Trends

Suicidal ideation/attempt (27.3% of patients) and eating disorders (15.2%) were the most frequent categories. Both showed a significant upward trend that was robust in the primary patient-level analysis and in the episode-level sensitivity analysis (Table 1). A significant increase was also observed for non-suicidal self-harm and alcohol/substance use. The upward trend for mood disorder, significant at the episode level, was attenuated and no longer statistically significant after FDR correction in the patient-level analysis, and is therefore interpreted with caution. Estimates for the smallest categories (e.g., psychosis/delirium and conversion/functional disorder) are based on very few patients and should be regarded as unstable. Anxiety/panic disorder showed no significant temporal trend in the patient-level analysis (IRR 1.06, 95% CI 0.89–1.25; FDR-adjusted *p* = 0.586).

### 3.3. Sex and Age

**Figure 2:**
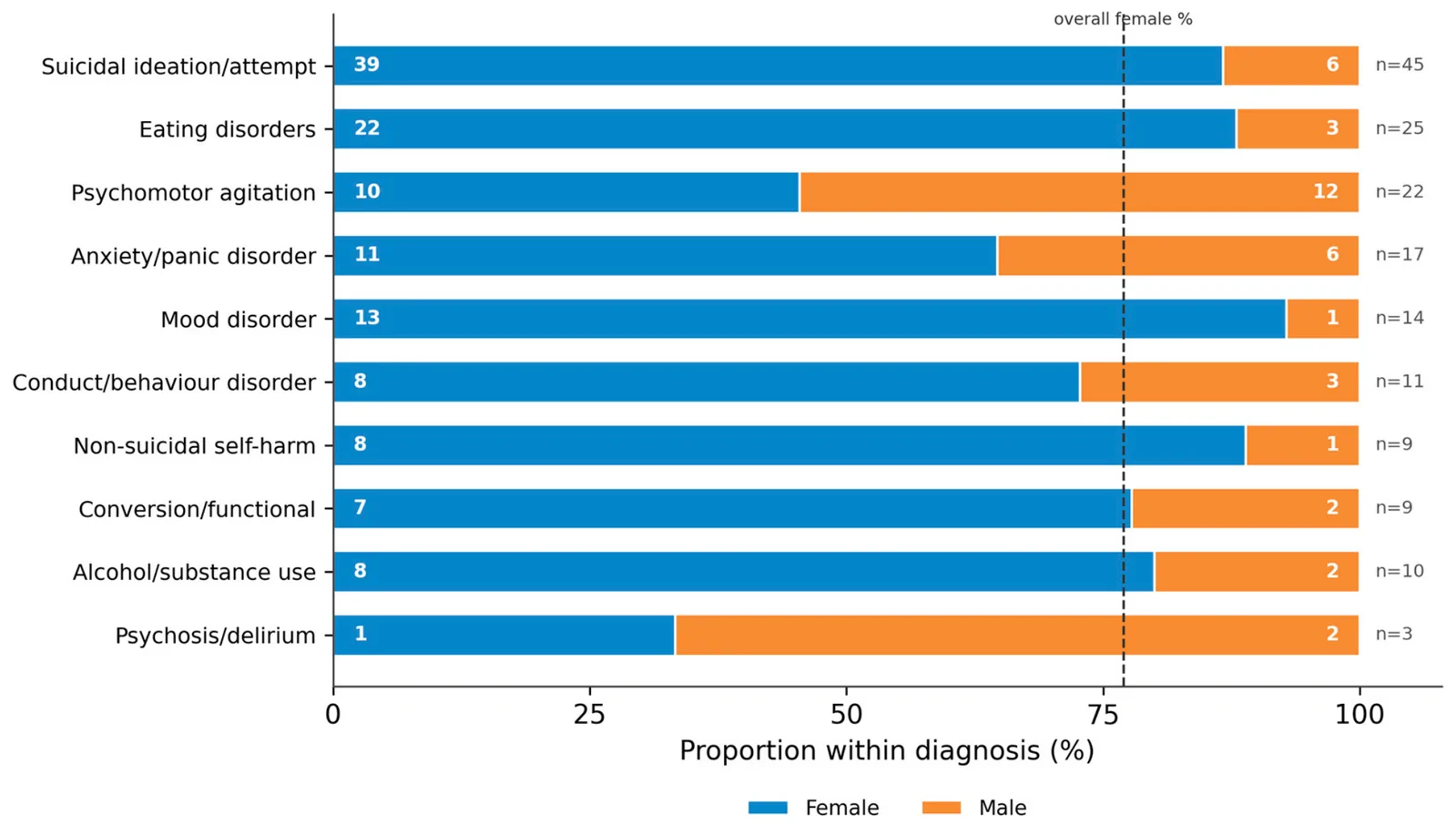
Sex distribution within each diagnostic group (unique patients). The dashed line marks the overall proportion of female patients.

**Figure 3:**
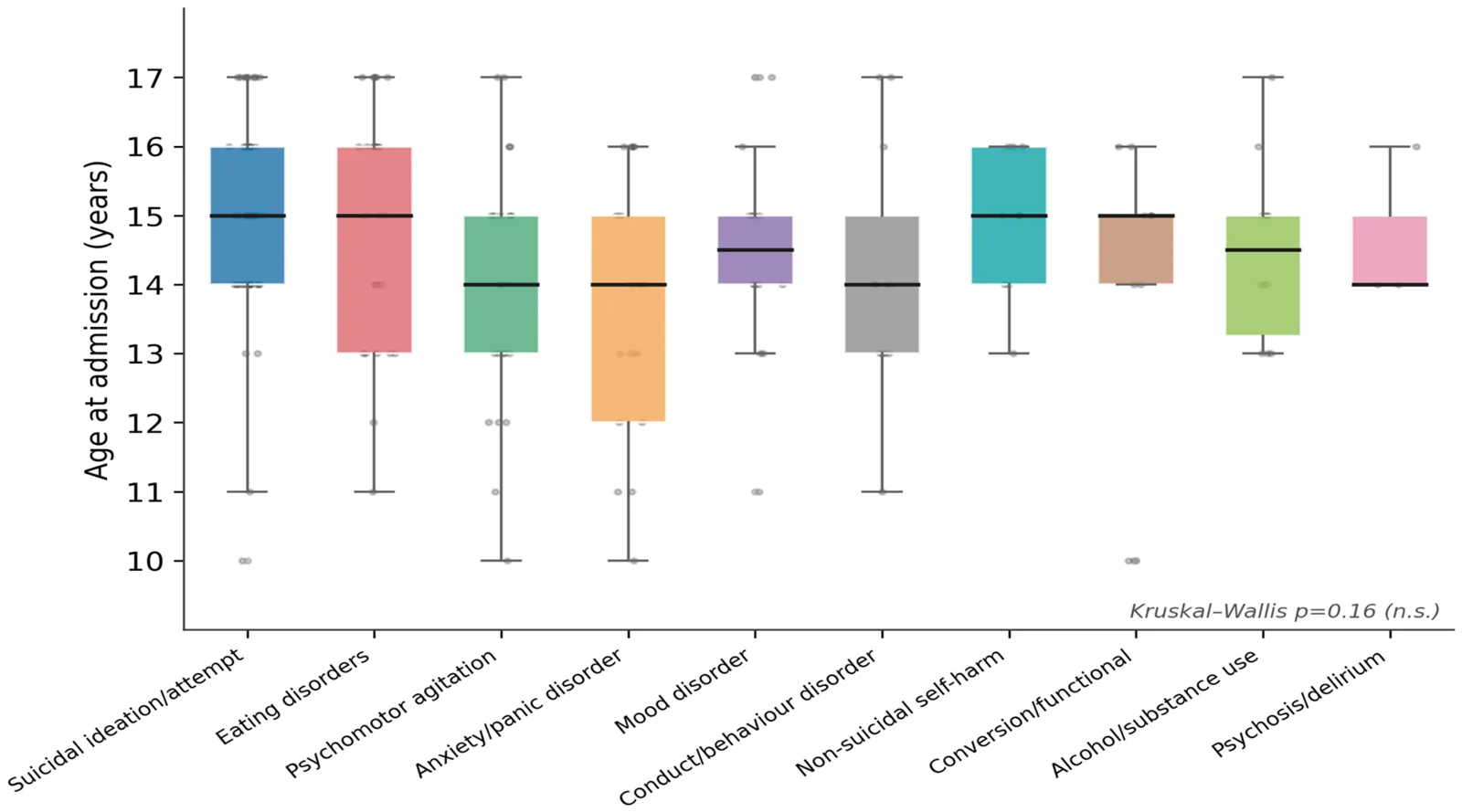
Age at admission by diagnosis (unique patients): box = interquartile range, line = median, whiskers = range, points = individual patients.

Diagnosis was significantly associated with sex in both the patient-level (chi-square = 23.98; *p* = 0.004; Cramér’s V = 0.38) and episode-level (chi-square = 25.43; *p* = 0.003; Cramér’s V = 0.35) analyses (Figure 2). Eating disorders and mood disorders occurred almost exclusively in females, whereas psychomotor agitation showed a markedly higher male proportion than the overall sample. In contrast, age at admission did not differ significantly across diagnostic groups in the primary analysis (Kruskal–Wallis *p* = 0.16; Figure 3); a nominally significant difference was present only at the episode level (*p* = 0.032) and is therefore not emphasized. Across the study period, age at admission showed a small but significant increase, corresponding to +0.14 years per calendar year (*p* = 0.010; Spearman ρ = 0.17, *p* = 0.027). Period-stratified descriptive data are shown in Supplementary Table S2. The female predominance was more pronounced in 2021–2025 (99/124, 79.8%) than in 2016–2020 (28/41, 68.3%), although the between-period difference in sex distribution was not statistically significant (*p* = 0.191; Table 2). Among patients with anxiety/panic disorder, females accounted for 3/7 cases (42.9%) in 2016–2020 and 8/10 (80.0%) in 2021–2025. However, the absolute numbers were small, and anxiety/panic disorder represented a lower proportion of all psychiatric patients in the later period (8.1% vs 17.1%).

### 3.4. Comparison Between the Two 5-Year Periods

**Figure 4:**
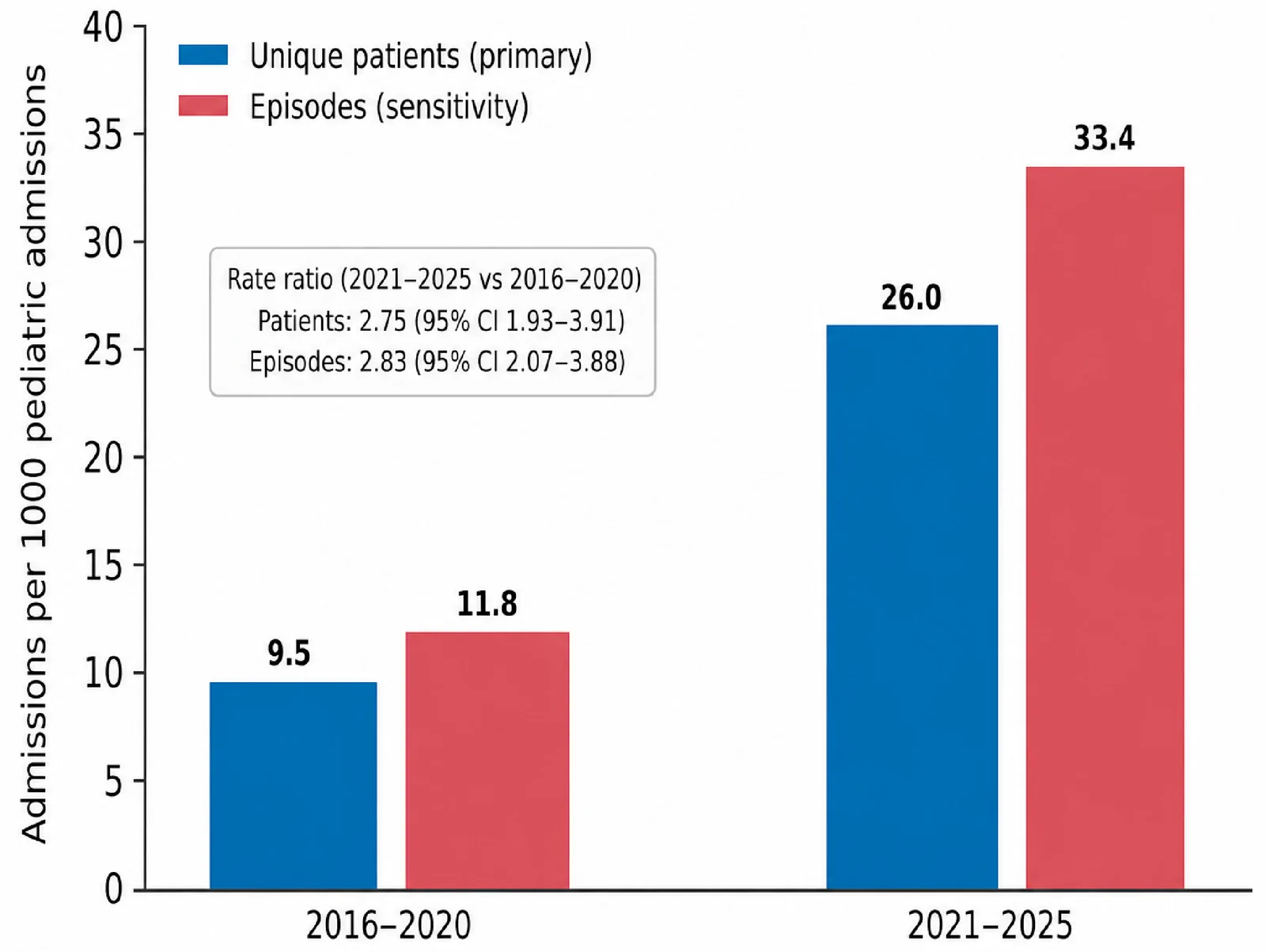
Admission rate per 1000 total pediatric admissions in the two 5-year periods, for unique patients (primary) and episodes (sensitivity).

In 2016–2020, 51 episodes were observed (11.78 per 1000 admissions), compared with 159 in 2021–2025 (33.39 per 1000), corresponding to a rate ratio of 2.83 (95% CI 2.07–3.88); the patient-level rate ratio was 2.75 (95% CI 1.93–3.91) (Table 2, Figure 4). Age at admission was higher in the second period, whereas the sex distribution did not differ significantly between periods. The relative diagnostic composition shifted toward suicidal ideation/attempt and eating disorders, although at the patient level this shift did not reach statistical significance.

### 3.5. Multivariable Analysis

In multivariable logistic regression (Table 3), suicidal ideation/attempt was independently associated with older age, while psychomotor agitation was strongly and inversely associated with female sex and showed a relative decrease over time, which reached statistical significance only in the episode-level analysis. No independent significant associations emerged for eating disorders or mood disorder. These null findings should be interpreted considering the limited number of outcome events, particularly for mood disorder (*n* = 14) and eating disorders (*n* = 25), which reduced model precision. Model results were consistent between the patient-level and episode-level analyses. Model fit was modest overall (McFadden pseudo-R^2^ 0.03–0.05 for suicidal ideation/attempt, eating disorders, and mood disorder) and higher for psychomotor agitation (0.14 at the patient level and 0.16 at the episode level). Given the limited number of events in some models, these estimates should be interpreted as exploratory.

### 3.6. Readmissions

Twenty-seven patients (16.4%) were hospitalized more than once, accounting for 45 readmissions (72 episodes in total; Table 4). These readmissions clustered in the most severe diagnostic categories: suicidal ideation/attempt (16), eating disorders (8), mood disorder (5), psychomotor agitation (4), anxiety/panic disorder (3), conduct/behavior disorder (3), non-suicidal self-harm (3), psychosis/delirium (2), and conversion/functional disorder (1). Because the primary analyses use each patient’s first admission only, these repeated admissions do not contribute to the patient-level estimates.

## 4. Discussion

According to our data, psychiatric hospitalizations of adolescents in a general pediatric ward increased markedly between 2016 and 2025, with a clear acceleration from 2021 onward. The most relevant feature was not only the trend itself but its magnitude: about three quarters of admissions occurred in the 2021–2025 period, with an approximately threefold increase in the admission rate compared with 2016–2020. Importantly, these findings were confirmed when the individual patient, rather than the hospitalization episode, was used as the unit of analysis, directly addressing the concern that repeated admissions might inflate the observed trends. The temporal increase also remained significant after accounting for annual variation in overall pediatric admission volume, indicating that it was not explained solely by changes in ward activity.

The added value of this single-center experience lies less in re-demonstrating the post-2020 rise—now well documented [3,4,5,6,7,8]—than in characterizing it within a general pediatric ward. However, the temporal coincidence with the COVID-19 pandemic should not be interpreted as evidence of a causal relationship. In settings where dedicated neuropsychiatric beds are scarce, the pediatric ward becomes a de facto safety net for acute adolescent psychiatric presentations, and its admissions provide a sentinel signal of the most severe component of adolescent distress. The observation that the 27 patients with repeated admissions clustered in suicidal ideation/attempt and eating disorders reinforces this interpretation: recurrence concentrated in the conditions of greatest clinical severity, a pattern with direct implications for continuity of care after discharge.

From a diagnostic perspective, the increase was driven mainly by suicidal ideation/attempt and eating disorders, both of which increased significantly and robustly. The prominence of suicidal ideation/attempt confirms that pediatric hospitalization increasingly intercepts the most acute component of adolescent psychological distress, in line with studies describing rising emergency and inpatient presentations for suicidality, self-harm, and internalizing disorders [4,5,14,15]. The number of patients hospitalized for eating disorders increased markedly in the later period, consistent with Italian studies documenting increased hospitalizations and referrals for eating disorders in developmental age during the pandemic period [16,17].

Placed alongside the international literature, our trends converge in direction with data from the United States [3,5,14], France [4,15], Finland [6], Spain [7], Denmark [18], and Italy [8,9,16,17,19], all describing rising acute and inpatient mental-health care use among young people, with a disproportionate impact on female and older adolescents. Our estimates are not directly comparable in magnitude, given differences in health-care organization and in the hospitalization setting; the value of the comparison lies in the consistency of the direction rather than in the equivalence of the rates.

The association between diagnosis and sex was confirmed at both the patient and episode levels, with a strong female predominance overall—particularly for eating and mood disorders—and a distinctively higher male proportion for psychomotor agitation. By contrast, we deliberately refrain from emphasizing differences in age across diagnostic groups, since this difference was not significant in the primary patient-level analysis. The modest but significant increase in age at admission over time, together with the concentration of the most severe diagnoses in mid-to-late adolescence, suggests that the greatest need for inpatient care emerges mainly in this age range.

Anxiety/panic disorder deserves separate consideration. Although the later-period cases were more frequently female in descriptive terms, this category did not show a significant temporal increase and accounted for a smaller share of patients in 2021–2025. Moreover, because each admission was assigned to a single dominant diagnosis, anxiety symptoms occurring alongside suicidal crises, mood disorders, or eating disorders may not have been captured as separate anxiety/panic admissions.

Our findings have organizational implications. Where child neuropsychiatric inpatient beds and community resources are limited, general pediatric wards increasingly care for patients with substantial psychiatric and psychosocial complexity, and pediatric hospitalization may serve as a sentinel indicator of severe adolescent suffering. The results support strengthening integrated pathways among pediatrics, child neuropsychiatry, emergency care, and community services, with particular attention to suicidal crises, eating disorders, and post-discharge continuity of care [13].

### Limitations

Several limitations should be acknowledged. First, the single-center design limits generalizability. Second, although we prioritized the individual patient as the unit of analysis and ambiguous cases were discussed collegially, the assignment of complex presentations to a single dominant diagnostic category still entails subjectivity and possible misclassification, and formal inter-rater reliability was not assessed. Third, our analyses are based on admission counts and on rates per 1000 total pediatric admissions, not on population-based incidence. Although the temporal trend remained significant after adjustment for annual pediatric admission volume, changes over time in referral pathways, service availability, admission thresholds, or local organization may have contributed to the observed trends, so conclusions about disease burden should be interpreted with caution. Fourth, several diagnostic categories were small, and the corresponding trend and regression estimates are consequently unstable and exploratory. Finally, the study captured ward admissions only and did not include psychiatric presentations managed in the emergency department without admission, which may underestimate the overall demand for acute care [14,19]. For these reasons, comparisons with the international literature should be read as convergence in direction rather than full equivalence of estimates [4,6,15].

## 5. Conclusions

This single-center series shows that psychiatric hospitalizations among adolescents admitted to a general pediatric ward increased substantially over the 2016–2025 decade, with a clear gradient from 2021 and a predominant contribution from suicidal ideation/attempt and eating disorders. These findings, robust to analysis at the patient level, support the view that general pediatric wards increasingly intercept a clinically severe share of adolescent mental distress and underline the need for stronger integrated pathways between pediatrics, child neuropsychiatry, emergency care, and community services, together with earlier, age- and sex-sensitive preventive strategies.

## Figures and Tables

**Table 1:** Diagnostic distribution and diagnosis-specific temporal trends (2016–2025), at the patient level (primary) and episode level (sensitivity).

Diagnostic Category	Patients *n* (%)	IRR/yr (95% CI) †	*p* (FDR)	Episodes *n* (%)	IRR/yr (95% CI) †	*p* (FDR)
Suicidal ideation/attempt	45 (27.3%)	1.19 (1.06–1.32)	0.011	61 (29%)	1.25 (1.13–1.37)	<0.001
Eating disorders	25 (15.2%)	1.28 (1.09–1.50)	0.011	33 (15.7%)	1.27 (1.11–1.45)	0.003
Psychomotor agitation	22 (13.3%)	1.02 (0.88–1.18)	0.824	26 (12.4%)	0.99 (0.86–1.13)	0.838
Anxiety/panic disorder	17 (10.3%)	1.06 (0.89–1.25)	0.586	20 (9.5%)	1.03 (0.88–1.20)	0.775
Mood disorder	14 (8.5%)	1.25 (1.02–1.54)	0.064	19 (9%)	1.27 (1.06–1.52)	0.021
Conduct/behavior disorder	11 (6.7%)	1.23 (0.98–1.54)	0.108	14 (6.7%)	1.23 (1.00–1.50)	0.071
Non-suicidal self-harm	9 (5.5%)	1.73 (1.17–2.54)	0.019	12 (5.7%)	1.44 (1.11–1.87)	0.021
Conversion/functional disorder	9 (5.5%)	1.32 (1.01–1.74)	0.072	10 (4.8%)	1.20 (0.95–1.51)	0.166
Alcohol/substance use	10 (6.1%)	1.44 (1.08–1.92)	0.031	10 (4.8%)	1.44 (1.08–1.92)	0.025
Psychosis/delirium	3 (1.8%)	1.28 (0.81–2.01)	0.364	5 (2.4%)	1.59 (1.00–2.52)	0.071
**Total**	**165 (100%)**	—	—	**210 (100%)**	—	—

† IRR, incidence rate ratio per calendar year estimated using Poisson regression; CI, confidence interval; FDR, false discovery rate. *p*-values were adjusted using the Benjamini–Hochberg procedure; bold indicates *p* < 0.05.

**Table 2:** Comparison between the two 5-year periods (2016–2020 vs 2021–2025).

Variable	2016–2020	2021–2025	*p*
Primary analysis—unique patients			
Admissions, *n*	41	124	—
Female sex, *n* (%)	28 (68.3%)	99 (79.8%)	0.191
Age, years—median (IQR)	14 (13–15)	15 (14–16)	0.036
Rate per 1000 admissions	9.47	26.04	RR 2.75 (1.93–3.91)
Sensitivity analysis—hospitalization episodes			
Admissions, *n*	51	159	—
Female sex, *n* (%)	37 (72.5%)	132 (83.0%)	0.150
Age, years—median (IQR)	14 (13–15)	15 (14–16)	0.011
Rate per 1000 admissions	11.78	33.39	RR 2.83 (2.07–3.88)

Rates are expressed per 1000 total pediatric admissions in the period (denominators: 4328 and 4762). RR: rate ratio. *p* from chi-square (sex) and Mann–Whitney (age).

**Table 3:** Multivariable logistic regression for four selected diagnoses (predictors: sex, age, year centered on 2016).

Outcome/Predictor	OR (95% CI)—Patients	*p*	OR (95% CI)—Episodes	*p*
Suicidal ideation/attempt (events: 45 pt/61 ep)				
Female sex	2.27 (0.86–5.97)	0.097	1.67 (0.71–3.93)	0.235
Age (per year)	1.28 (1.02–1.6)	**0.031**	1.24 (1.02–1.52)	**0.035**
Year (per year, from 2016)	0.94 (0.81–1.08)	0.396	1.02 (0.9–1.15)	0.747
Eating disorders (events: 25 pt/33 ep)				
Female sex	2.25 (0.63–8.06)	0.211	2.47 (0.71–8.63)	0.155
Age (per year)	1.16 (0.88–1.52)	0.288	1.17 (0.91–1.5)	0.216
Year (per year, from 2016)	1.06 (0.88–1.27)	0.535	1.04 (0.89–1.21)	0.608
Psychomotor agitation (events: 22 pt/26 ep)				
Female sex	0.21 (0.08–0.55)	**0.002**	0.21 (0.08–0.52)	**<0.001**
Age (per year)	0.88 (0.67–1.16)	0.360	0.83 (0.65–1.07)	0.161
Year (per year, from 2016)	0.84 (0.7–1)	0.056	0.81 (0.69–0.96)	**0.013**
Mood disorder (events: 14 pt/19 ep)				
Female sex	4.17 (0.52–33.19)	0.177	4.64 (0.6–36.12)	0.143
Age (per year)	0.98 (0.7–1.36)	0.893	1 (0.74–1.35)	0.996
Year (per year, from 2016)	1.04 (0.82–1.31)	0.755	1.05 (0.86–1.28)	0.612

OR, odds ratio; CI, confidence interval; pt, patients; ep, episodes. Events per model are reported to allow assessment of stability; models with few events are exploratory. McFadden pseudo-R^2^ is reported in the text; bold indicates *p* < 0.05.

**Table 4:** Distribution of the number of hospitalizations per patient over the study period.

Hospitalizations per Patient	Patients, *n* (%)	Episodes, *n* (%)
1 (single admission)	138 (83.6%)	138 (65.7%)
2	16 (9.7%)	32 (15.2%)
3	5 (3.0%)	15 (7.1%)
4	5 (3.0%)	20 (9.5%)
5	1 (0.6%)	5 (2.4%)
Patients with ≥2 admissions	27 (16.4%)	72 (34.3%)
Total	165 (100%)	210 (100%)

Percentages in the patient column are calculated on 165 unique patients; percentages in the episode column are calculated on 210 hospitalization episodes. Readmissions refer to admissions occurring after each patient’s first hospitalization during the study period.

## Data Availability

Anonymized data from patients were used for this analysis. Calculations made for statistical analysis are described in the text. Further inquiries can be directed to the corresponding author.

## Supplementary Materials

The following supporting information can be downloaded at https://www.mdpi.com/article/10.3390/pediatric18040111/s1: Table S1: Annual total pediatric admissions, unique psychiatric patients, psychiatric hospitalization episodes, and corresponding rates per 1000 admissions, 2016–2025; Table S2: Diagnostic distribution by sex in the two study periods at the individual-patient level.

## References

[1] World Health Organization (2025). Mental Health of Adolescents. https://www.who.int/news-room/fact-sheets/detail/adolescent-mental-health.

[2] UNICEF (2021). The State of the World’s Children 2021: On My Mind. https://www.unicef.org/reports/state-worlds-children-2021.

[3] Arakelyan M., Freyleue S., Avula D., McLaren J.L., O’mAlley A.J., Leyenaar J.K. (2023). Pediatric mental health hospitalizations at acute care hospitals in the US, 2009–2019. JAMA.

[4] Gutierrez-Sacristan A., Serret-Larmande A., Hutch M.R., Sáez C., Aronow B.J., Bhatnagar S., Bonzel C.-L., Cai T., Devkota B., Hanauer D.A. (2022). Hospitalizations associated with mental health conditions among adolescents in the US and France during the COVID-19 pandemic. JAMA Netw. Open.

[5] Overhage L., Hailu R., Busch A.B., Mehrotra A., Michelson K.A., Huskamp H.A. (2023). Trends in acute care use for mental health conditions among youth during the COVID-19 pandemic. JAMA Psychiatry.

[6] Gyllenberg D., Marttila M., Sund R., Jokiranta-Olkoniemi E., Sourander A., Gissler M., Ristikari T. (2018). Temporal changes in the incidence of treated psychiatric and neurodevelopmental disorders during adolescence: An analysis of two national Finnish birth cohorts. Lancet Psychiatry.

[7] Gonzalez-Fraile E., Soriano V., Ramos J.M., Gallego L., Berzosa-Grande M.P., López-Ibor M.I., Chiclana-Actis C., Mestre-Bach G., Faraco M., Pinargote H. (2025). Two decades of hospital admissions for adolescents with depression in Spain. J. Affect. Disord..

[8] Amianto F., Arletti L., Baietto C., Davico C., Migliaretti G., Vitiello B. (2022). Trends in admissions to a child and adolescent neuropsychiatric inpatient unit in the 2007–2017 decade: How contemporary neuropsychiatry is changing in Northwestern Italy. Eur. Child Adolesc. Psychiatry.

[9] Bozzola E., Ferrara P., Spina G., Villani A., Roversi M., Raponi M., Corsello G., Staiano A. (2022). The pandemic within the pandemic: The surge of neuropsychological disorders in Italian children during the COVID-19 era. Ital. J. Pediatr..

[10] Istituto Nazionale di Statistica (ISTAT) (2025). Health Indicators on Mental Health by Age and Sex. https://www.istat.it/.

[11] Istituto Superiore di Sanità-Epicentro (2023). HBSC Italia 2022: National Data. https://www.epicentro.iss.it/hbsc/indagine-2022-nazionali-convegno-8-febbraio-2023.

[12] Società Italiana di Neuropsichiatria dell’Infanzia e dell’Adolescenza (SINPIA) Increase in Psychiatric Hospitalizations Among Minors (Press Release, 8 November 2021). https://sinpia.eu/.

[13] Ministero della Salute (2019). Linee di Indirizzo sui Disturbi Neuropsichiatrici e Neuropsicologici dell’Età Evolutiva. https://www.salute.gov.it/.

[14] Bommersbach T.J., McKean A.J., Olfson M., Rhee T.G. (2023). National trends in mental health-related emergency department visits among youth, 2011–2020. JAMA.

[15] Fond G., Pauly V., Brousse Y., Llorca P.-M., Cortese S., Rahmati M., Correll C.U., Gosling C.J., Fornaro M., Solmi M. (2025). Mental health care utilization and prescription rates among children, adolescents, and young adults in France. JAMA Netw. Open.

[16] Cappelletto P., Luca L., Taddei B., Taddei S., Nocentini A., Pisano T. (2024). COVID-19 pandemic impact among adolescents with eating disorders referred to Italian psychiatric unit. J. Child Adolesc. Psychiatr. Nurs..

[17] Giacomini G., Elhadidy H.S.M.A., Paladini G., Onorati R., Sciurpa E., Gianino M.M., Borraccino A. (2022). Eating disorders in hospitalized school-aged children and adolescents during the COVID-19 pandemic: A cross-sectional study of discharge records in developmental ages in Italy. Int. J. Env. Res. Public Health.

[18] Bliddal M., Rasmussen L., Andersen J.H., Jensen P.B., Pottegård A., Munk-Olsen T., Kildegaard H., Wesselhoeft R. (2023). Psychotropic medication use and psychiatric disorders during the COVID-19 pandemic among Danish children, adolescents, and young adults. JAMA Psychiatry.

[19] Ferro V., Averna R., Murciano M., Raucci U., Cristaldi S., Musolino A.M.C., Pontillo M., Della Vecchia N., Labonia M., Pisani M. (2023). Has anything changed in the frequency of emergency department visits and the profile of the adolescent seeking emergency mental care during the COVID-19 pandemic?. Children.

