# Supplementary material for: Hospitalizations of Adolescents with Psychiatric Disorders in a Pediatric Unit: A 10-Year Retrospective Study
Source: Pediatr Rep. 2026 Aug 10;18(4):111. doi: 10.3390/pediatric18040111 (PMC13516791; doi:10.3390/pediatric18040111)
Supplement: Supplementary file 1 [file pediatrrep-18-00111-s001.zip › pediatrrep-4454480-supplementary.pdf]

Table S1. Annual total pediatric admissions, unique psychiatric patients, psychiatric hospitalization episodes, and corresponding rates per 1000 admissions, 2016–2025.

Year	Total pediatric admissions	Unique psychiatric patients, n	Patient-level rate per 1000 admissions	Psychiatric hospitalization episodes, n	Episode-level rate per 1000 admissions
2016	858	6	6.99	8	9.32
2017	921	5	5.43	9	9.77
2018	894	8	8.95	10	11.19
2019	983	8	8.14	10	10.17
2020	672	14	20.83	14	20.83
2021	739	28	37.89	34	46.01
2022	891	25	28.06	29	32.55
2023	1058	18	17.01	27	25.52
2024	1093	15	13.72	20	18.30
2025	981	38	38.74	49	49.95
**Total**	**9090**	**165**	**18.15**	**210**	**23.10**

Unique patients were assigned to the calendar year of their first psychiatric admission during the study period. Episode-level counts include all psychiatric hospitalizations, including readmissions. Rates are expressed per 1000 total admissions to the pediatric ward.

Table S2. Diagnostic distribution by sex in the two study periods at the individual-patient level.

**Diagnostic category**	**2016–2020**		**2021–2025**	
	**Female, n (%)**	**Male, n (%)**	**Female, n (%)**	**Male, n (%)**
Suicidal ideation/attempt	9 (100.0%)	0 (0.0%)	30 (83.3%)	6 (16.7%)
Eating disorders	4 (100.0%)	0 (0.0%)	18 (85.7%)	3 (14.3%)
Psychomotor agitation	4 (44.4%)	5 (55.6%)	6 (46.2%)	7 (53.8%)
Anxiety/panic disorder	3 (42.9%)	4 (57.1%)	8 (80.0%)	2 (20.0%)
Mood disorder	2 (66.7%)	1 (33.3%)	11 (100.0%)	0 (0.0%)
Conduct/behaviour disorder	3 (75.0%)	1 (25.0%)	5 (71.4%)	2 (28.6%)
Non-suicidal self-harm	1 (100.0%)	0 (0.0%)	7 (87.5%)	1 (12.5%)
Alcohol/substance use	1 (100.0%)	0 (0.0%)	7 (77.8%)	2 (22.2%)
Conversion/functional disorder	1 (50.0%)	1 (50.0%)	6 (85.7%)	1 (14.3%)
Psychosis/delirium	0 (0.0%)	1 (100.0%)	1 (50.0%)	1 (50.0%)
**Total**	**28 (68.3%)**	**13 (31.7%)**	**99 (79.8%)**	**25 (20.2%)**

Values are n (%), with percentages calculated within each diagnostic category and study period. Analyses are based on each patient's first psychiatric admission. Owing to sparse cells, period-specific sex distributions are presented descriptively.
